# The psychological well-being of children orphaned by AIDS in Cape Town, South Africa

**DOI:** 10.1186/1744-859X-5-8

**Published:** 2006-07-19

**Authors:** Lucie Cluver, Frances Gardner

**Affiliations:** 1 Department of Social Policy and Social Work, University of Oxford, UK; 2 Cape Town Child Welfare, Gatesville, Cape Town, South Africa

## Abstract

**Background:**

An estimated 2 million children are parentally bereaved by AIDS in South Africa. Little is known about mental health outcomes for this group.

**Methods:**

This study aimed to investigate mental health outcomes for urban children living in deprived settlements in Cape Town. 30 orphaned children and 30 matched controls were compared using standardised questionnaires (SDQ) on emotional and behavioural problems, peer and attention difficulties, and prosocial behaviour. The orphan group completed a modified version of a standardised questionnaire (IES-8), measuring Post-Traumatic Stress symptoms. Group differences were tested using t-tests and Pearson's chi-square.

**Results:**

Both groups scored highly for peer problems, emotional problems and total scores. However, orphans were more likely to view themselves as having no good friends (p = .002), to have marked concentration difficulties (p = .03), and to report frequent somatic symptoms (p = .05), but were less likely to display anger through loss of temper (p = .03). Orphans were more likely to have constant nightmares (p = .01), and 73% scored above the cut-off for Post-Traumatic Stress Disorder.

**Conclusion:**

Findings suggest important areas for larger-scale research for parentally-bereaved children.

## Background

An estimated 24.8% of South Africa's population are HIV+, with 4.7 million infected by 2001 [1]. Numbers of children parentally bereaved by AIDS in South Africa are expected to rise from 1.1 million in 2003, to 3.1 million by 2010 [2], peaking at 5.7 million in 2015. Even with the proposed full administration of anti-retroviral therapy, estimates remain at 1.15 million maternal orphans by 2015 [3].

Orphaned children in South Africa have traditionally been cared for within the extended family [4], often by elderly grandparents [5]. There are concerns that this support system is weakening as orphan numbers and HIV prevalence increase [6]. There are few reliable data on numbers of orphans living in non-kin fostering arrangements, institutions, child-headed households and as streetchildren [7].

Most work on orphans concentrates on basic needs. This is understandable as AIDS-affected households are characterised by economic deprivation, often exacerbated by medical costs [6]. Orphans frequently lack sufficient food, shelter, schooling and medical care, and are at risk of abuse and economic exploitation [8-10].

There is little available research, but increasing concern, regarding the psychological well-being of orphans in Africa. Children orphaned by AIDS are exposed to multiple stressors which may compound and complicate the grieving process. They may have cared for and witnessed the death of parent/s with a debilitating illness, loss of bodily functions, and sometimes AIDS-related mental illness [11,12]. AIDS can cause multiple losses, for example of mother, father and perinatally-infected younger siblings. Caregivers of orphans have been found to suffer poor psychological health themselves [13,14]. South African orphans report that stigma and secrecy surrounding AIDS causes social isolation, bullying, shame, and a lack of opportunity to openly discuss their loss [15]. Poor levels of AIDS-related knowledge and communication can lead to children being ignorant of the cause of death, or fearing that they will also be infected [16].

Searches of literature on mental health for orphaned children found 10 unpublished studies and 6 published studies to date. Of these, 6 lacked a control group and 9 compared children parentally bereaved by AIDS with some kind of controls. There may be difficulties translating US studies to an African context, with differing support systems and characteristics of HIV-infected groups. There is also danger in assuming that studies conducted in one part of Africa are transferable to the South African context. This is the first quantitative study known to be completed in South Africa.

### Studies in Africa

Sengendo and Nambi (1997) [17] interviewed 169 orphans under the education sponsorship of World Vision in rural Uganda, and a comparison group of 24 non-orphans. On a non-standardised scale, orphans experienced more depression than non-orphans. Makame et al. (2002) in urban Tanzania, used a scale based on the Rand Inventory and items from the Beck Depression inventory, and found increased internalising problems and suicidal ideation in orphans (n = 41) compared with non-orphans (n = 41). Manuel et al. [13] used a questionnaire based on Makame et al in rural Mozambique, and found orphans (n = 76) more likely than controls (n = 74) to be depressed and bullied, and less likely to have a trusted adult or friend. Carers of orphans showed more depression and less social support. Poulter (1996) interviewed carers in 22 Zambian households with orphans, 66 with HIV+ parents, and 75 controls. On the Rutter scales, carers reported orphans as more unhappy and worried than children with HIV+ parents, who were more so than controls. No clear link was found between distress and poverty, and there was no evidence of conduct problems [18]. Wild, Flisher, Laas and Robertson [19], in the Eastern Cape of South Africa, used standardised questionnaires with orphans (n = 80) and both other-orphan and non-orphan control groups, and found that children orphaned by causes other than AIDS reported more depression, anxiety and lower self-esteem than non-orphans, with children orphaned by AIDS falling between the two groups. In rural Uganda, Atwine, Cantor-Graae and Banjunirwe [20], used standardised questionnaires (Beck Youth Inventory) with 115 orphaned children and 110 matched non-orphaned children. Orphans had greater risk of anxiety, depression and anger.

Two further, unpublished, studies found in Africa, were unable to be accessed. A mention of Gelman [21], in Zimbabwe, reports only the finding that existing Western psychometric tools could not be validated. An interim report of Elmore-Meegan et al (ongoing) in Kenya, describes a multi-centre study using an adaptation of the Achenbach CBCL. Preliminary results suggest more depression and stress amongst orphans.

Non-controlled studies in Africa include Foster, Makufa, Drew, Mashumba & Kambeu, (1997) in rural Zimbabwe. In focus-groups, orphaned children (n = 40) reported anxiety, fear, stigmatisation, depression and stress. Nampanya-Serpell [22] used structured interviews with families of rural and urban Zambian orphans, and found emotional disturbance related to separation from siblings and increased family size. Volle et al. [23] interviewed 788 orphans in Zambia. 89% reported unhappiness, and 19% running away from their new homes. Makaya et al. (2002), used clinical interviews with 354 Congolese orphans, and found 20% experiencing psychological difficulties, including depression, anxiety and irritability (34%), fugue, offending and hyperactivity (27%), and PTSD (39%).

### Studies in the USA

In New York, orphans (n = 30) reported more peer and externalising problems on standardised instruments, than children with HIV+ parents (n = 29) [24]. Another New York study [25,26], using longitudinal assessments with standardised instruments, found that bereaved children reported more emotional distress and problem behaviours than children whose parents were alive and HIV+.

The Family Health Project [27,28] used standardised instruments with 20 maternal orphans and 40 non-orphans. Affected children were assessed pre-orphanhood, and at 6 months after bereavement. Children of HIV+ mothers showed more internalising and externalising problems, and lower cognitive and social competence than controls. 6 months after orphanhood, there were non-significant improvements, and authors suggested that this may be related to increased stability and wealth amongst new caregivers. However, at 2 years, orphans showed higher levels of internalising (but not externalising) problems. Hirsch [29] compared 16 maternal orphans by AIDS and 18 'other' orphans, and found higher depression, anxiety and conduct problems, on standardised scales, amongst children orphaned by means other than AIDS.

The limited research suggests the possibility that orphans may be experiencing higher levels of internalising and, to a lesser extent, externalising problems. These studies, combined with qualitative research on orphan well-being, also hint at more specific areas of difficulty. Makaya (2002) reported high levels of PTSD amongst Congolese orphans, and studies have linked childhood PTSD to traumatic parental death, especially the witnessing of that death [30-32] Orphans have reported difficulty concentrating at school, due to worries, sadness or tiredness [27,33]. Concentration problems may be linked to post-traumatic stress, as could anecdotal reports of recurrent nightmares. Literature also suggests somatic symptoms [34], which may be a useful indicator of distress amongst children in South Africa [35]. Finally, friendship difficulties related to stigma have been found in both qualitative [9,33] and quantitative studies [13,36].

This study was conducted at the request of the Cape Town Child Welfare Society. Little is known about the effects of orphanhood in urban South Africa. Studies show multiple stressors of violence and poverty common to all township children, but there may be additional difficulties for parentally bereaved children in these communities. Furthermore, there is a clear need for further studies using both matched non-affected control groups, and standardised instruments, to test hypotheses suggested by the literature to date.

### Hypothesis

We hypothesised that children who were orphaned by AIDS would show a higher incidence of psychological difficulties than a non-orphaned control group from the same community. Specific areas derived from the literature include difficulty with concentration, friendships, traumatic and somatic symptoms.

## Methods

Data collection took place in the Cape Flats around Cape Town, South Africa, in both formal and 'informal' (shack-based) settlements, between 2002 and 2003. Orphans were compared with children who had not experienced a parent dying of AIDS. The mental health of HIV+ children is an important area of research, but this study focused on children who were not known to be HIV+.

This study compares orphaned to non-orphaned children. However, there is ongoing debate in South Africa around these distinctions. In contexts such as social services and financial provision, there are strong arguments for interventions targeting the wider group of 'Orphans and Vulnerable Children' [37] or universally targeting poor children [38]. Whilst recognising the validity of these arguments, it is equally important to have a secure evidence base for understanding the effects of different factors on child mental health. This study aims to contribute to an understanding of whether orphanhood by AIDS has a psychological impact, compared to non-orphaned children, within deprived communities.

### Participants

The participants were 60 African children aged 6 to 19, living in varying care arrangements, in the settlements of Old Crossroads, Nyanga, Langa, Guguletu, Philippi, Blue Downs and Browns Farm. Thirty controls were matched by neighborhood, ethnicity, age and gender. Children were recruited from a number of services, including the Cape Town Child Welfare Society (children awaiting and in foster placements), shelters for streetchildren and children's homes. In order to access orphans who were not receiving any welfare services, children were also recruited through schools and community centres.

### Procedure

Children were interviewed in their place of residence or school. Privacy was maintained as much as possible, although this was sometimes difficult in overcrowded households. The research was conducted in the child's first language, Xhosa or English. Questionnaires were translated and blind back-translated. Blindness was partially achieved for interviewers, who were unaware of whether the child was an orphan until the final page of the questionnaire. This contained the specific 'orphan' questions, and was blank for non-orphans. The questionnaire took 15–20 minutes to complete.

### Ethics

This is a highly sensitive area and care was taken not to distress the children. Interviewers were Xhosa or English-speaking social workers or careworkers trained in working with children with HIV/AIDS. Due to low literacy levels, information and consent leaflets were also discussed verbally, and interviewers explained to children that they could refuse to participate in the research at any point. Following previous studies (Makame, 2002, Manuel, 2002, Poulter, 1996), HIV/AIDS was not mentioned in research materials. Ethical approval was obtained from Oxford University and Cape Town Child Welfare Society.

### Measures

The Strengths and Difficulties Questionnaire [39], an internationally well-validated screening tool for child emotional and behavioral difficulties, was read aloud to children. Additional questions addressed difficulties identified in earlier studies, including peer relationships, experience of violence, hunger and school attendance (Makame et al. 2002; Manuel 2002) and demographic questions. The orphan group only were given a brief questionnaire relating to PTSD-type symptoms, using items from the shortened Impact of Events Scale (IES-8) [40]. Since the IES-8 requires that the child has experienced an identified stressful event, and this was only available for orphans, it was not given to the control group.

The SDQ includes subscales for prosocial behavior, hyperactivity/attentional, emotional, conduct and peer problems. Scores >90th percentile predict substantially raised probability of independently diagnosed psychiatric disorders (Goodman 2001). The SDQ has been translated into 51 languages, and extensively validated in many Western and developing countries (Mullick & Goodman, 2001), but not in South Africa.

Stallard et al. [41] found the IES-8 to correctly identify two-thirds of children with diagnosed PTSD and borderline conditions. Smith et al. [42] compared the IES-8 used in the UK with factor analysis of the IES-8 used in Bosnia, and found the same structure of intrusion and avoidance, with similar factor loadings for each item in both groups. Although we could find no data for the use of IES-8 in South Africa, a Bosnian study suggests that intrusion and avoidance symptoms can be comparable for children in different cultures. Following Winje and Ulvik [32], the scaling of the IES was changed from a four-point scale (0–1–3–5) to a three-point scale (0 = no degree, 1 = some degree, 2 = high). Thus, the mean subscale scores in this study should be prorated (× 2.5) to compare symptom severity levels in other studies using the IES. Winje and Ulvik report internal reliability of .72, intrusion, .75, avoidance.

### Analysis

Group comparisons used Pearson's χ^2 ^for categorical data, and t-tests for comparing mean scale scores. Where literature strongly suggested that a particular item may differ between groups, we analyzed individual items. Caution is needed in analysing single items, and we attempted to avoid multiple comparisons by only testing hypotheses generated from the literature.

## Results and discussion of results

### Demographic factors

Table 1 shows no significant group differences on demographic questions. Almost half of all children went to bed hungry 1 or more days per week. Most attended school full-time (26 non-orphans and 23 orphans). Experience of violence was similar, with slightly more non-orphans reporting seeing or experiencing violence. Children reported similar levels of 'trusted adults', although 17% of children reported none.

**Table 1:** Demographic Factors

		Orphans n = 30	Non-orphans n = 30	P value	χ^2^
Age	*Mean*, (range)	11 (7–19)	12 (6–19)	0.42	7.2
		SD 3.0	SD 2.8		
Gender	males	19	14	0.19	1.7
	females	11	16		
School	Days attended *Mean*, (range)	4.3 (0–6)	4.8 (3–5)	0.30	7.2
Hunger	Days hungry *Mean*, (range)	1.3 (0–7)	1.1 (0–7)	0.79	3.2
Trusted adult	'somewhat' or 'very true'	83%	83%	0.35	2.1
Violence	'somewhat' or 'very true'	44%	57%	0.12	4.3

### Strengths and difficulties questionnaire (table 2)

**Table 2:** Scores for Orphans and Non-Orphans on SDQ

	Orphans n = 30 *Mean*, (SD)	Non-orphans n = 30 *Mean*, (SD)	P value t-test (2-tailed)
Pro-social total score	8.8 (1.9)	7.8 (2.2)	0.35 ns
Conduct problems total score	2.4 (1.9)	2.8 (2.2)	0.55 ns
Peer problems total score	3.5 (2.6)	4.2 (2.1)	0.28 ns
Hyperactivity total score	2.6 (2.0)	3.2 (1.4)	0.16 ns
Emotional problems total score	4.2 (2.7)	3.5 (2.4)	0.35 ns

#### Prosocial behavior

There were no differences between orphan and non-orphan groups in total scores for pro-social behavior, or for any individual questions.

#### Conduct problems

There were no group differences in total conduct problem scores. One item showed a difference, namely that non-orphans were more likely to report getting angry and losing their temper than orphans (χ^2 ^6.8, p = .03). One of the fears relating to the unknown psychosocial effects of orphanhood in South Africa was the prospect of anti-social behavior due to orphans 'raised without supervision' [43]. This presumption has been challenged [44], and this study finds no evidence of increased self-reported conduct problems amongst orphans.

#### Peer problems

There were no group differences in peer problems. However, only one orphan felt that they definitely had a good friend (χ^2 ^9.4, p = .01). Orphans were more likely to perceive themselves as not having any good friend (χ^2 ^9.4, p = .02) (Table 3).

**Table 3:** Individual items from the SDQ whose importance was suggested by earlier studies

	Orphans	Non-orphans	P value	χ^2^
Extreme difficulty concentrating	(n = 29)	(n = 29)		
	10 (34%)	3 (10%)	0.03	4.9
Very frequent Nightmares	(n = 30)	(n = 30)		
	13 (45%)	4 (13%)	0.01	7.1
Definite lack of close friendship	(n = 29)	(n = 30)		
	29 (97%)	19 (66%)	0.002	9.4
Very frequent somatic symptoms	(n = 30)	(n = 30)		
	12 (40%)	5 (17%)	0.05	3.7

The lack of differences in overall peer problems is encouraging when compared to evidence of stigma and discrimination affecting orphans [13,17,45]. However, the difference in the single item 'I have one good friend or more' shows that 97% of orphans perceived themselves as having no close friend. It is possible that this reflects stigma and myths around proximity to AIDS [33]. It may also reflect PTSD-type symptoms such as detachment, avoidance and difficulties in forming close relationships [46]. An association was found for orphans between PTSD 'caseness' (cut-off score below/above 17) and endorsement of the item 'children don't want to be friends with me' (χ^2 ^6.1, p = .05). The high proportion of children who met criteria for borderline or abnormal peer problems (58%) suggest high levels of overall need in both orphans and non-orphans.

#### Hyperactivity

There were no group differences in total hyperactivity scores, with a slight trend towards non-orphans being more hyperactive than orphans. However, orphans were more likely to experience extreme difficulty in concentrating (i.e. endorsing this difficulty 'all the time') (χ^2 ^4.9, p = .03) (Table 3). The overall scale suggests that other aspects of hyperactivity are not seen as a problem by AIDS orphans, and motor overactivity was not apparent from anecdotal observation. It is possible that problems with concentration are related to distress or Post-Traumatic Stress Symptoms, rather than hyperactivity [47]. Other studies have found concentration difficulties amongst orphans [27,36,48] and lower educational achievement [17,27].

#### Emotional problems

There were no group differences in overall emotional problems. On the somatic item from the emotional scale, orphans were more likely to report recurrent 'stomach-aches, headaches or sickness' (χ^2 ^3.7, p = .05). The lack of difference in the total emotional problems score may indicate that orphans do not experience higher levels of anxiety and depression than non-orphans. However, studies undertaken in the Cape Flats have found very high overall levels of internalising distress amongst children in general [49], and this may make it more difficult to isolate sub-groups within this population.

Qualitative and clinical somatisation studies suggest that the group difference on the somatic question might indicate a way of expressing distress for orphaned children. Another possible interpretation of the somatic item is that these are not psychosomatic but actual illnesses, as studies report insufficient medical care of orphans due to poverty, discrimination and misattribution of illnesses to HIV infection.

### Post-Traumatic Stress

Questions relating to Post-Traumatic Stress were given only to orphans. According to the protocol for IES-8, PTSD questions were asked in relation to the death: 'how you have felt...about the death of your parent/s'. It is likely that most children remembered the event, as their mean age when orphaned was 8 years (SD 3.2) and mean duration of orphanhood was 3.5 years (SD 2.5).

#### PTSD 'caseness' in orphans

**Figure 1:**
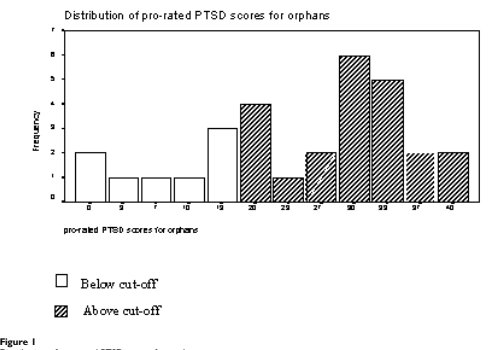
Distribution of pro-rated PTSD scores for orphans.

Using a cut-off score of 17 (found to correctly identify 47 of 49 UK children with a clinical diagnosis of PTSD; [50]), 73.3% of the orphan group fulfilled the criteria for suffering from PTSD (figure 1). This is an extremely high level, but it is important to be cautious in using 'caseness' definitions drawn largely from Western countries

One question on the PTSD scale was possible to incorporate into the questionnaire given to both orphans and non-orphans : 'I have nightmares or sad dreams'. It is normal for children to experience some nightmares, but constant and recurrent nightmares are one defining symptom of PTSD (Yule, 2001). Within the groups, more orphans (45%) than non-orphans (13%) suffered from nightmares 'all the time' (χ^2 ^7.1, p = .01) (Table 3). The non-orphan group did not answer PTSD questions, but the group differences for the only shared question raises the possibility that PTSD-type symptoms may be more common in orphans than non-orphans.

#### Correlates of PTSD

There were no differences in mean PTSD scores for boys (26.5 SD 10.5) vs. girls (19.4 SD 12.8). There were no associations between PTSD score and age of child (r = -.05), or age of child when their parent died (r = 0.14) and number of years the child had been orphaned (r = -0.15). There was an association between PTSD scores and SDQ emotional problems (p = .04).

## General discussion

This study found no evidence of higher levels of self reported emotional and behavioral problems in orphans, using the SDQ. This must be understood within the context of deprivation affecting both orphaned and non-orphaned children in the urban settlements around Cape Town. For example, both groups showed high levels of exposure to violence and hunger. Norms established for the SDQ [51] may be useful to indicate where there are high levels of need, although caution must be used as there are no SDQ norms available for Africa. However, studies such as Fazel and Stein [52] have used British SDQ norms to assess refugee children from multiple areas of origin. When comparisons are made with British norms, both the orphaned and non-orphaned groups showed higher levels of difficulty in peer problems, emotional problems and total SDQ scales (1–2, 0.5 and 0.5 SDs higher, respectively). The orphan group showed 3× higher 'caseness' levels than British norms for peer problems. Given the cautions raised about 'caseness', we might tentatively suggest that there are high levels of some problems for orphaned and non-orphaned children living in the deprived townships studied.

However, on some items suggested by earlier studies to be particularly relevant to orphan mental health, there were differences, including apparently high levels of PTSD-type symptoms. Orphans were less likely than non-orphans to have a good friend, more likely to have difficulty concentrating and to report somatic symptoms. There was no evidence of conduct or behavioral problems amongst orphans.

PTSD symptoms among orphans may be related to a number of stressors. Death of a parent from AIDS could be highly traumatic: the vast majority of AIDS victims in South Africa remain at home, which are often informal housing containing a whole extended family, and children may perform roles of carers. For example, government leaflets instruct on washing soiled bedclothes of AIDS victims [53]. Thus many children witness the slow, painful death of a parent in degrading circumstances. The intermittent nature of the disease, stigma and secrecy around the death, the move into foster care, into a child-headed household, or onto the streets, could all potentially contribute to trauma for children.

## Conclusion

### Limitations of the study

Limitations of the current study should be noted. This research cannot show whether the problems identified are related to orphanhood in general, rather than orphanhood by HIV/AIDS. Future studies may need a comparison group of children orphaned by reasons other than AIDS. This design could be methodologically challenging in the urban South African context: cause of death is frequently hard to ascertain and many reported non-AIDS deaths are due to AIDS. In addition, further exploration is needed of the traumatic effects of orphanhood due to non-AIDS causes such as violent crime.

The most striking findings of this study are in the PTSD scale, but this is the only part of the questionnaire which was not also given to the control group of non-orphans. However, the IES-8 scale requires an identifiable event from which to measure intrusion and avoidance symptoms. There was no such easily identifiable event for the non-orphan group, which would not have also been experienced by orphans. Surveys of PTSD amongst children in the Cape Flats suggest high overall levels of symptoms [54,55]. None of these studies have yet distinguished between orphaned and non-orphaned children, and this is an area which clearly requires further research.

Further limitations include the reliance on child self-report of symptoms, and it is recognised that multiple informants are preferable when assessing children [56]. However, we had serious methodological difficulties in identifying and accessing suitable informants for many children in the orphan group. These included children who were living with new and unknown foster parents, with unwell caregivers, in shelters for streetchildren, and in child-headed households.

Blind back-translation of the questionnaire, information and consent forms found the quality of the Xhosa version to be good. However, the population studied was different from those in which the scales used had been developed and standardised, therefore knowledge of reliability and validity for this population is limited. Cross-cultural measures of mental health always contain the possibility of difference in meaning between researchers and participants. For example, it is possible that the measures of self-reported depression in the SDQ were not ideally suited to Xhosa culture. The single item analyses must be read with caution, although all were generated from earlier literature. Moreover, cut-offs used for both the SDQ and PTSD scales are problematic, although useful in indicating high levels of need. These limitations mean that the findings from this study should be treated cautiously.

Practical difficulties in this study included stigma and secrecy around HIV/AIDS, which often delayed identification of and access to affected families and children. High levels of illiteracy, overcrowded dwellings and crime resulted in non-ideal research settings.

However, the limitations of this study should be considered within the context of its strengths. This is the first study in South Africa, and one of the first in Africa, to use standardised questionnaires and a matched, non affected control group in measuring psychological well-being of children orphaned by AIDS. The findings suggest important areas for larger-scale research into the mental health of orphaned children. Despite the limited evidence, these findings suggest that children orphaned by AIDS may have unmet psychological needs. The finding of strikingly high PTSD-type symptoms in this study indicate that this should be a key area for research and intervention. Further research is also needed to identify risk and protective factors for orphans, and into the effects of differing care arrangements, as rising orphan numbers may lead to an increase in child-headed households and streetchildren.

Currently, very few organisations provide psychosocial support for children who are parentally bereaved by AIDS, and only a small minority of children receive support. The findings of this and other studies suggest that there is a need for effective interventions to reach a larger proportion of orphaned children. Such interventions must be sensitive to the differing cultural norms and political agendas around HIV/AIDS in South Africa. They must also function within the scarce resources available in communities supporting orphans of the AIDS epidemic.

## Competing interests

The author(s) declare that they have no competing interests.

## Authors' contributions

LC carried out the fieldwork. FG and LC participated in the design of the study, performed the statistical analysis and drafted the manuscript. Both authors read and approved the final manuscript.
